# The Monocrotaline Rat Model of Right Heart Disease Induced by Pulmonary Artery Hypertension

**DOI:** 10.3390/biomedicines12091944

**Published:** 2024-08-23

**Authors:** Anna Maria Krstic, Timothy L. M. Jones, Amelia S. Power, Marie-Louise Ward

**Affiliations:** 1 Department of Physiology, University of Auckland, Auckland 1142, New Zealand; a.krstic@auckland.ac.nz (A.M.K.); timothy.4.jones@cuanschutz.edu (T.L.M.J.); a.power@auckland.ac.nz (A.S.P.); 2 Division of Cardiology, Anschutz Medical Campus, University of Colorado, Aurora, CO 80045, USA

**Keywords:** pulmonary artery hypertension, right ventricular hypertrophy, right heart failure, monocrotaline, cardiomyocyte, excitation–contraction coupling

## Abstract

Pulmonary artery hypertension (PAH) is characterised by increased pulmonary vascular resistance (PVR) resulting in elevated pressure in the pulmonary artery supplying the pulmonary circulation. Disease of the right ventricle (RV) often manifests as a result of PAH placing excessive pressure on the right side of the heart. Although a relatively rare disease in humans, the impact of sustained PAH is severe, with poor outcomes even in treated individuals. As PAH develops, the blood flow is restricted through the pulmonary arteries and the right ventricle hypertrophies due to the increased strain of pumping blood through the pulmonary circulation. With time, RV hypertrophy progresses to right heart failure, impacting the supply of blood to the left ventricle and systemic circulation. Although right heart failure can currently be treated, it cannot be cured. There is therefore a need for more research into the physiological changes that cause the heart to fail under pressure overload. This review aims to evaluate the monocrotaline (MCT) rat model of PAH as a means of studying the cellular mechanisms associated with the development of RV hypertrophy and right heart failure.

## 1. Introduction

The pulmonary vasculature is a low-pressure system capable of accommodating the same cardiac output as ejected from the left ventricle. It is therefore a highly compliant system, able to respond to large changes in the blood volume supplied by the right ventricle (RV) in response to physiological increases in venous return [1]. The RV is a relatively thin-walled chamber of the heart in comparison to the more bulky left ventricle, with fewer cardiomyocytes contributing to each heartbeat. This makes the RV less able to cope with acute increases in afterload, in comparison to the left ventricle, reducing the RV ejection fraction. Increased pulmonary vascular resistance (PVR) and pulmonary artery pressure (PAH) means that the right ventricle must work harder to pump blood throughout the pulmonary capillaries. If sustained, RV hypertrophy develops in response to an increased afterload. Initially, the hypertrophy enables the RV to contract more forcefully, maintaining the flow through the pulmonary vasculature. However, over time, the sustained elevation in PVR progresses towards right heart failure. As this develops, the supply of oxygenated blood to the left ventricle and systemic circulation becomes compromised. Common symptoms of heart failure are then observed. In patients, symptoms such as breathlessness, fatigue, angina, syncope and fluid retention occur [2]. Right heart failure is observed in 3–9% of acute heart failure patients admitted to hospital, with a high patient mortality rate [3].

In humans, pulmonary hypertension (PH) is classified as a mean pulmonary arterial pressure of ≥20 mmHg [4] and can be caused by a number of different pathologies. These include bone morphogenetic protein receptor II mutations [5], chronic obstructive pulmonary disease [6] and chronic thromboembolism PH [7]. Secondary PH and RV hypertrophy can also develop as a result of left heart disease, following the development of pulmonary congestion, which increases the afterload experienced by the RV [8,9,10]. It is a rare disease in humans that develops in response to a diverse number of aetiologies affecting the pulmonary vasculature [11], and it is frequently observed in patients with heart valve disease [4]. Irrespective of the origins of the disease, increased pulmonary vascular resistance (PVR) places an increased afterload on the right ventricle, causing RV hypertrophy. With time, this progresses to RV heart failure, which limits the filling of the LV with oxygenated blood, reducing the cardiac output.

The response of the right side of the heart to pulmonary hypertension is also known as “cor pulmonale”, a Latin term meaning “pulmonary heart”. It describes alterations in the structure and function of the RV that are caused by a primary disorder of the respiratory system resulting in pulmonary hypertension. Cor pulmonale is not used to describe right ventricular disease that develops secondary to left-sided heart failure or congenital heart disease affecting the RV.

Currently, despite recent technological advances and an increased understanding of the anatomy and physiology of RV heart failure, there are relatively few treatment strategies and evidence-based interventions available for its effective treatment [12]. A number of animal models have been developed to better understand the progression of RV disease resulting from PH (for a review, see [13]). Models commonly used include exposure to chronic hypoxia, with or without the addition of Sugen, an endothelial growth factor receptor antagonist; the induction of an acutely increased RV afterload following pulmonary arterial banding [14]; and the monocrotaline rat model discussed in this review. Infections resulting from the blood parasite *Schistosoma* are a very common cause of PAH in humans worldwide, and mice with the parasite develop varying degrees of PAH over weeks to months [15]. A mouse model of pneumonectomy has also been developed, with the aim of reproducing some of the developmental disorders resulting in PAH [16]. Importantly, the use of animal models of PH enables a better understanding of the mechanisms driving the progression from RV hypertrophy to failure [13].

## 2. The Monocrotaline Rat Model of Pulmonary Artery Hypertension

The monocrotaline (MCT) rat model has featured in hundreds of peer-reviewed publications investigating the mechanisms associated with PAH [17]. Its popularity is largely due to its simplicity, non-invasiveness, consistency and affordability when compared to other animal models, such as pneumonectomy, chronic hypoxia or pulmonary artery banding [18].

### 2.1. Historical Perspective

Monocrotaline is derived from *Crotalaria spectabilis,* a member of the pea family of plants native to the Indo-Malaysian region. Although the toxicity of the *Crotalaria spectabilis* plant was well known, it was not until 1890 that the alkaloid source of the toxicity was discovered [19]. The alkaloid was first extracted from the seeds of *Crotalaria spectabilis* by Neal, Rusoff and Ahmann (1935), who suggested the name monocrotaline (commonly abbreviated to MCT) [20]. Further study of this molecule by Adams and Rogers (1938) discovered it to be an 11-membered macrocyclic pyrrolizidine alkaloid with the formula C_16_H_23_NO_6_ [21].

Early research into the toxicity of MCT focused on its hepatotoxic effects until Lalich and Merkow (1961) noted that the ingestion of *Crotalaria spectabilis* seeds, or extracted MCT, induced pulmonary arteritis in rats [22,23]. They observed that female Sprague-Dawley rats who ingested either the seeds of the *Crotalaria spectabilis* plant or extracted MCT displayed severe pulmonary arteritis, whereas there was no effect in rats who were fed with *Crotalaria spectabilis* seeds that had undergone MCT extraction [23]. The observation by Kay et al. in 1967 that MCT consistently caused PAH in female Wistar rats motivated investigations into the MCT rat as a model of PAH [24]. The MCT rats also displayed RV hypertrophy and failure that mirrored that of PAH in humans, with the primary cause of death (~70%) in patients with PAH attributed to RV disease [25].

### 2.2. Development of MCT-Induced Toxicity

The “MCT syndrome” is characterised by pulmonary vascular lesions, PAH, RV hypertrophy and hepatic veno-occlusive disease [26]. MCT is metabolised by cytochrome P-450 3 A in the liver to monocrotaline pyrrole (MCTP) [27,28], which was identified as the cause of the pneumotoxicity [29,30]. Bruner et al. (1986) established that the biological half-life of MCTP in the blood of rats was ~5 s, allowing sufficient time for MCTP to reach the lung capillary beds after production in the liver [31,32]. While MCT is cleared from the body very quickly, MCTP is maintained in the red blood cells in sufficient quantities to cause damage to endothelial cells for up to 24 h following MCT administration [26,33,34]. Although the mechanism of action of MCT remains partially unexplained, the cellular targets of MCTP are primarily pulmonary arterial endothelial cells [24]. This fits the PH classification of Group 1, induced by pre-capillary vascular remodelling in response to MCT [11].

Within the first 24 h after MCT administration, there is evidence of mononuclear inflammatory cell infiltration into the adventitial sheaths of the small pulmonary arteries and veins [35]. This is associated with oedema and the initial contraction of the pulmonary smooth muscle cells in the small pulmonary arteries [35]. Pulmonary arterial endothelial cell damage and inflammation is followed by the significant proliferation of pulmonary smooth muscle cells in the following days, extending into the normally non-muscular pulmonary arteries [36,37]. Ultimately, these effects culminate in the narrowing of the lumen of the small pulmonary arteries and increasing resistance, and thus pressure, within the pulmonary artery. These luminal changes in the arteries provide the pressure overload that drives the development of RV hypertrophy and progression to right heart failure (i.e., cor pulmonale) [37]. Evidence of right ventricular inflammation has been observed in patients with right heart disease, and the inflammatory response is now accepted as a key part of cardiovascular disease progression [38]. It is therefore also likely to contribute to MCT-induced RV hypertrophy and failure, in addition to the pulmonary inflammation induced by monocrotaline administration [36,37].

### 2.3. Response to MCT Administration in Rats

MCT-induced cor pulmonale was first described by Kay et al. in 1967, as *Crotalaria spectabilis*’ oral administration consistently increased the ratio of RV–body weight in female Wistar rats [24]. The subcutaneous injection of a single dose of MCT to young adult rats is commonly used as a non-invasive means of inducing pulmonary hypertension that progresses to right ventricular hypertrophy and heart failure over a period of 4–6 weeks. The rate of RV disease development following MCT injection is dose-dependent, with higher doses (100 mg kg^−1^ body weight and above) producing significantly greater RV hypertrophy in a shorter period of time than lower doses [39]. A low MCT dose of 30 mg kg^−1^ body weight can be used for the study of compensated RV hypertrophy as these animals do not show signs of progression to heart failure up to 12 weeks [40].

The choice of rat strain is also important for the induction of cor pulmonale [41,42,43,44]. The two most common rat strains used for the MCT model are Wistar and Sprague-Dawley rats. However, Wistar rats appear to develop a more severe response to MCT than Sprague-Dawley rats, which exhibit a 15% lower mortality rate [41]. There are also sex differences following MCT injection. Mature female rats have an increased median survival time compared to mature male rats when administered a high dose of MCT (although this sex difference is not present in immature rats) [42]. Female rats also exhibit a reduced cardiac response to MCT administration, showing non-significant changes in RV weight at a stage when male rats have an 85% increase in RV weight, 5 weeks after treatment with 60 mg kg^−1^ body weight of MCT [44]. The observed sex differences in the progression of MCT-induced PAH and RV disease in young adult rats are likely to be a result of beneficial female hormones affecting lung development and maturation [45].

**Figure 1:**
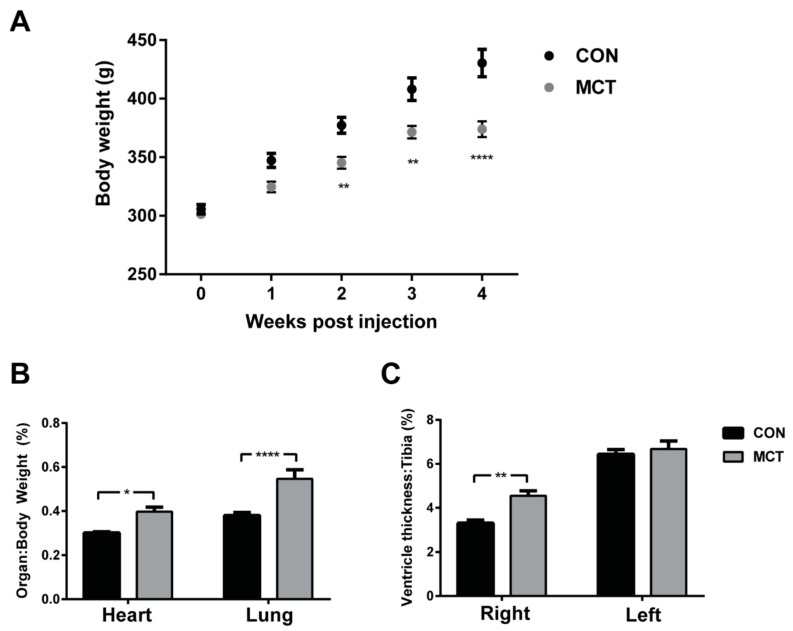
Evidence of right ventricular hypertrophy in MCT rats. Body weight (g) of rats following injection of 60 mg kg^−1^ monocrotaline (MCT; *n* = 10; grey) or saline (CON; *n* = 10; black) prior to the excision of the heart and lungs at 4 weeks post-injection (**A**). Wet weights of the heart and lungs normalised to the rat body weight (**B**). Following the dissection of the hearts, the thickness of each ventricle free wall was taken at the mid-line of the heart between the base and the apex and measurements are shown normalised to the tibial length (**C**). Statistical significance between CON and MCT is denoted by * *p* < 0.05, ** *p* < 0.01, **** *p* < 0.0001 using two-way ANOVA with multiple comparisons [49].

The most commonly used monocrotaline concentration for the study of cor pulmonale is 60 mg kg^−1^ body weight, administered via intraperitoneal or subcutaneous injection [46]. This dose will usually produce RV hypertrophy (~2 weeks post-injection [47]) and RV failure approximately 4–5 weeks post-injection [48]. The regular monitoring of MCT rats post-injection is required as RV hypertrophy progresses rapidly to failure. This includes noting any physical signs of deterioration, such as the loss of normal curiosity, poor grooming, a hunched posture and rapid breathing, as well as the daily monitoring of their body weight, food consumption and water intake. Figure 1A shows the growth of a cohort of age-matched male Wistar rats following the subcutaneous injection of either monocrotaline (MCT, 60 mg kg^−1^) or sterile saline (CON). Notably, the growth (indexed as body weight) of MCT-injected rats was significantly attenuated from 2 weeks post-injection in comparison to saline-injected controls, prior to any other overt symptoms in the MCT rats.

## 3. Myocardial Changes in Response to MCT

MCT-injected rats are frequently studied at two different endpoints: (i) during compensated RV hypertrophy prior to the onset of heart failure (e.g., [50,51,52]) and (ii) at end-stage heart failure (e.g., [53,54,55]). Han et al. (2017) obtained in vivo recordings of the heart rate and blood pressure (systolic and diastolic) in male Wistar rats (300–325 g body weight) after the injection of either monocrotaline (MCT) or sterile saline (CON). Differences were observed between groups in the heart rate and blood pressure from 28 days post-injection, with significant differences by day 35, indicating the onset of right heart failure in MCT [55]. A more rapid transition to RV heart failure has been reported in male rats injected with 60 mg kg^−1^ MCT at a younger age (200 g body weight), with external signs of heart failure observed 18–26 days post-injection (e.g., [56]).

Our research group has focused on studying MCT rat hearts during compensated hypertrophy from days 28 to 35 post-injection [50,51,57]. At 4 weeks post-injection, MCT rats show clear evidence of RV hypertrophy (Figure 1B,C), before overt signs of heart failure. Knowledge of the early changes in the RV before progression to end-stage failure may help to identify novel treatment targets that prevent the transition to heart failure.

### 3.1. Compensated RV Hypertrophy

Morphometric data from MCT rats four weeks post-injection and their saline-injected controls (CON) is shown in Table 1. MCT rats had greater RV thickness–body weight (%) and greater lung weights relative to the CON rats, which are characteristic of RV hypertrophy [51]. Notably, signs of heart failure (i.e., prolonged QTc interval and hepatomegaly) were absent in MCT rats 4 weeks post-injection, although the lower body weight in comparison to the controls suggests progression towards right heart failure.

### 3.2. Cardiomyocyte Changes during Compensated RV Hypertrophy

**Figure 2:**
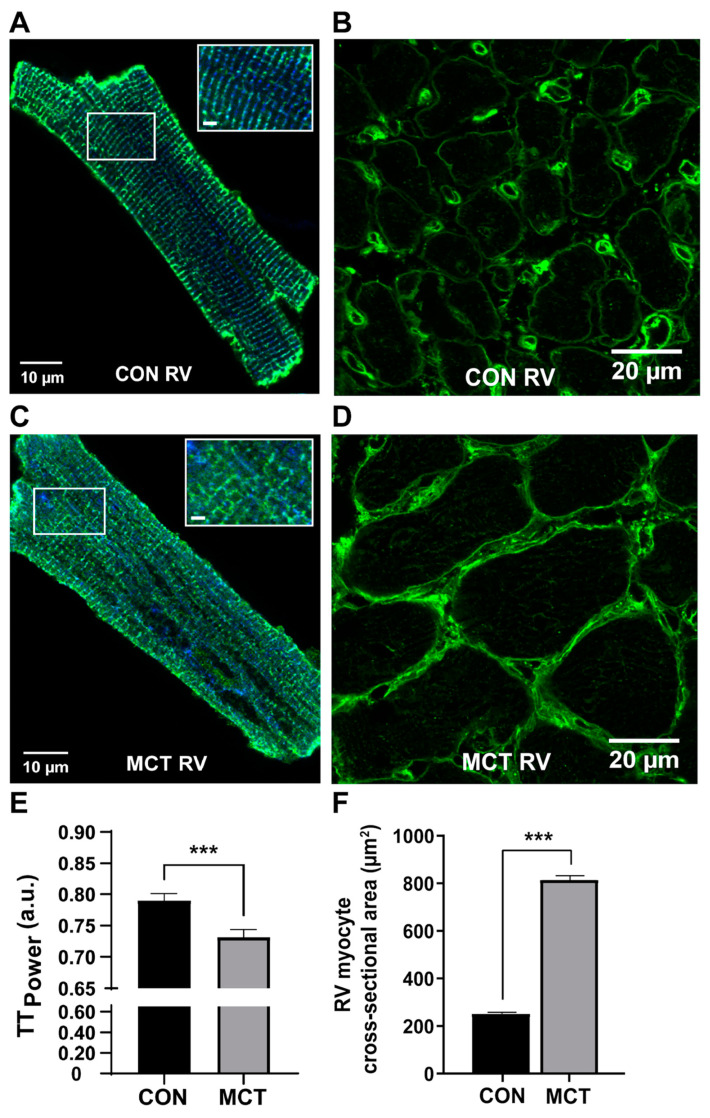
Evidence of RV hypertrophy 4 weeks post-injection. (**A**,**C**) show representative cardiomyocytes isolated from the RV of CON (**A**) and MCT (**C**) at 4 weeks post-injection, labelled for ryanodine receptors (RyR2, blue) and sarcolemmal marker Caveolin-3 (Cav-3, green). (**E**) shows the mean TT_Power_ value that represents t-tubule regularity in CON (black) and MCT (grey) RV cardiomyocytes. Results are expressed as mean ± SEM, *n =* 23 RV cardiomyocytes from *N* = 3 CON hearts and *n =* 18 RV cardiomyocytes from *N* = 3 MCT hearts. *** *p* < 0.001. (**B**,**D**) show representative confocal images of fixed right ventricular (RV) tissue sections from CON (**B**) and MCT (**D**) hearts, labelled with a sarcolemma and extracellular matrix marker (wheat germ agglutinin). (**F**) shows the mean ± SEM cross-sectional area from *N* = 3 CON and *N* = 3 MCT hearts. MCT RV myocytes (*n* = 108) had a larger cross-sectional area in comparison to CON RV (*n* = 160) myocytes (*** *p* < 0.001). Modified from [51].

Cardiomyocytes are the working cells of the heart, responsible for contracting with sufficient force to generate the required pressure to supply blood throughout the circulatory system. During compensated RV hypertrophy, prior to the onset of heart failure, the hypertrophic heart exhibits structural and functional alterations at the cellular level, which contribute to changes in whole heart function. At 4 weeks post-injection, the RV cardiomyocyte cross-sectional area was increased in MCT RV tissue relative to MCT LV and to RV and LV cardiomyocytes from control tissue, as illustrated for RV in Figure 2. The increase in the cardiomyocyte size was evidence of disease progression within the RV in the MCT rat hearts [51].

Mitochondria supply more than 95% of the ATP required to fuel the continuous cycles of contraction and relaxation in the heart [58]. As a result, mitochondria comprise about 30% of the healthy cardiomyocyte volume. The investigation of hypertrophied RV cardiomyocytes from MCT rat hearts at 4 weeks post-injection showed increased mitochondrial protein abundance, with no difference in mitochondrial respiration or membrane potential in comparison to the controls [52]. However, MCT myocytes had larger beat-to-beat changes in mitochondrial Ca^2+^ transients compared to control myocytes, suggesting that increased mitochondrial Ca^2+^ uptake is essential in matching the energy supply to the increased metabolic demands of the hypertrophied cardiomyocytes [52]. Following the transition from compensated hypertrophy to RV failure, the mitochondrial oxidative capacity is severely depressed [59]. This is particularly evident for complex-I-linked respiration [59], suggesting that a disrupted energy supply drives the transition to RV failure in MCT, with implications for myocardial efficiency as complex-I-linked respiration contributes more ATP per oxygen consumed. This has been demonstrated in trabeculae isolated from MCT-injected rats in RV failure [60], where the supra-basal efficiency was lower compared to saline-injected controls, determined using calorimetry [60].

## 4. Impact of MCT on Cardiomyocyte Excitation–Contraction Coupling

Right ventricular function has been studied in MCT-injected rat models in vivo and in a variety of isolated cardiac preparations. The flexibility of the preparation choice is a definite advantage of the MCT rat model, with researchers utilising ventricular trabeculae [44,50,55,60,61,62,63,64,65], isolated cells [66,67,68,69,70] and isolated–perfused whole hearts [55,69,70,71]. These studies have provided valuable functional insights into cardiomyocyte function as hearts transition to RV hypertrophy and eventually failure.

Cardiomyocyte excitation–contraction coupling is initiated by the cardiac action potential propagating across the surface sarcolemma and throughout the t-tubule system to ensure synchronous depolarisation throughout. In healthy cardiomyocytes, voltage-gated L-type Ca^2+^ channels, predominantly in the t-tubule sarcolemma, open and allow the influx of Ca^2+^ into the dyad during the Ca^2+^ current; for a review, see [72]. The Ca^2+^ influx in turn induces the further release of Ca^2+^ from the sarcoplasmic reticulum, or intracellular Ca^2+^ store, in a process known as calcium-induced calcium release (CICR), [73]). In this way, an approximately ten-fold increase in cytosolic Ca^2+^ is rapidly brought about, termed the “Ca^2+^ transient”. Ca^2+^ then diffuses to the contractile proteins, initiating cross-bridge cycling and force production.

### 4.1. Changes in Excitation–Contraction Coupling during Compensated RV Hypertrophy

In addition to increased contractile protein and cardiomyocyte enlargement, there was initially the upregulation of Ca^2+^ ion flux as a compensatory response of the hypertrophic myocardium, which enabled the RV to contract against the greater workload. Isolated RV myocytes from MCT rats 14 days post-injection had significantly longer action potential durations from day 14 onwards (becoming progressively longer with time post-injection) when compared to saline-injected controls [74], with a significantly increased L-type Ca^2+^ current density. However, this was not sustained, and, by 28 days post-injection, the Ca^2+^ transient amplitudes were reduced in MCT in comparison to the controls [50]. At this stage, with the administration of 60 mg kg^−1^ of body weight of MCT, the RV trabeculae from male Wistar rats showed a reduced Ca^2+^ transient amplitude, increased diastolic Ca^2+^ leakage, a reduced response to β-adrenergic stimulation and the significant disruption of the t-tubular system [50].

Confocal images of representative isolated RV cardiomyocytes at 4 weeks post-injection are shown in Figure 2A,C, with MCT cardiomyocytes showing evidence of a disrupted t-tubule structure. Similar t-tubule disruption was found in PFA-fixed RV trabeculae from MCT labelled with wheat germ agglutinin [50]. The t-tubule integrity is important for cardiomyocyte action potential propagation, enabling rapid and synchronised contraction [75]. These studies show that the disruption of t-tubules occurs early in the disease process, before RV failure is established.

The changes in cardiac excitation–contraction coupling observed in MCT-treated rats provide valuable insights into the mechanical and electrical functions of cardiomyocytes as they undergo hypertrophy and eventually failure. The physiological differences strongly depend on the stage of heart failure progression, which can be modified in this model depending on the dose of MCT, the amount of time post-administration and the age, strain and sex of the rats. The body weight of the rats at the time of MCT injection, rather than the age per se, is key to the rate of RV disease progression. For example, rats injected at ~350 g body weight do not develop heart failure until 5–6 weeks post-injection (e.g., [53]), whereas rats injected at ~200 g body weight transition to RV failure at around 3–4 weeks post-injection (e.g., [52]).

### 4.2. Beta-Adrenergic Response of MCT-Induced RV Hypertrophy

Sympathetic stimulation of the heart results in increased inotropic, lusitropic and chronotropic effects and is the physiological mechanism by which healthy hearts respond to increased stress. At 4 weeks post-injection, the β-adrenergic stimulation of RV trabeculae with 1 µM isoproterenol increased the Ca^2+^ transient amplitude and active stress in the controls but not in MCT, despite accelerating the Ca^2+^ transient decay in trabeculae from both groups [50]. During isoproterenol treatment, MCT trabeculae showed increased diastolic Ca^2+^ leakage, which may explain the blunted inotropic response to β-adrenergic stimulation. In addition, hypertrophic MCT RV myocytes also exhibited spontaneous Ca^2+^ release events during β-adrenergic stimulation with isoproterenol, which was subsequently recovered by partial sarcoplasmic reticulum ATPase inhibition. This was, in part, due to the increased Ca^2+^ store content shown by large caffeine-induced Ca^2+^ transients in RV myocytes from MCT vs. CON [52]. Krstic et al., (2023) also showed an increased mitochondrial abundance and beat-to-beat mitochondrial calcium uptake in MCT RV myocytes, which is a compensatory response to match the ATP supply to the increased energetic demands of the hypertrophic heart [52].

Patients with pulmonary hypertension and right-sided heart failure show RV-specific desensitisation to β-adrenergic stimulation [76]. Isolated–perfused MCT hearts in the sinus rhythm, prior to the onset of right heart failure, showed no significant increase in heart rate in response to 0.03 µM isoproterenol added to the perfusate, as well as a reduced percentage increase in LV-developed pressure in comparison to control hearts. MCT hearts in the sinus rhythm also showed a smaller change in the pressure–time integral at the peak of the isoproterenol response in comparison to controls [77]. These data suggest that MCT rats also exhibit desensitisation to β-adrenergic stimulation, as seen in human disease.

## 5. Limitations of the Model and Conclusions

The MCT model does not perfectly replicate the pathology of human PAH, as it does not display the plexiform lesions (complex glomeruloid-like vascular structures) [78] commonly seen in human PAH [26]. Additionally, Ruiter at al., (2013) showed that a smaller dose of MCT (40 mg kg^−1^ of body weight) produced RV hypertrophy and PAH early in the disease’s progression; however, the pulmonary vascular resistance and RV systolic pressure returned to control levels 8 weeks after MCT administration [79]. While the ventricular hypertrophy was maintained, the pressure overload was relieved due to pulmonary vascular remodelling [79]. This does not occur when using a standard dose of MCT (60 mg kg^−1^ of body weight) as animals usually die from right heart failure at 4–6 weeks post-administration [48]. While this may limit the applicability of the MCT model to the study of human PAH, these differences do not discount the MCT model as a model of cor pulmonale as it remains a non-invasive and consistent method of raising the pulmonary arterial pressure in order to induce RV hypertrophy and right heart failure.

Pulmonary artery banding is an alternative animal model used to study the response to PAH. A previous study in rats showed that, 6 weeks after fitting a band that partially occluded the pulmonary artery, the RV systolic pressure was increased to 60 mm Hg, with a twofold increase in the RV mass, which was comparable to the response observed in MCT-injected rats. However, the pulmonary artery banding rats had no signs of heart failure, unchanged RV volumes and systemic hemodynamics and increased RV contractility [80]. A comparison with the MCT-treated rats in the Hessel et al., 2006 study suggests that the effects of the pressure overload and the mechanisms underlying contractility and RV dilatation are substantially different between the two models [81].

In conclusion, the subcutaneous injection of a single dose of monocrotaline to young adult rats is a non-invasive means of inducing pulmonary hypertension that leads to right ventricular hypertrophy and heart failure over a period of 4–6 weeks. The MCT rat therefore provides an inexpensive, consistent and easily obtained model for the study of the transition from compensatory RV hypertrophy to RV failure. In addition, it has the added advantage of developing pulmonary artery hypertension over a period of days to weeks. While there are differences in the lung injury pattern in comparison to PAH in humans, the MCT rat model consistently reproduces experimental cor pulmonale with dose-dependent effects on the RV. Overall, it is a useful rat model for the study of the diseased right ventricle, providing valuable insights into the progression of RV disease from hypertension and hypertrophy to RV failure.

## Figures and Tables

**Table 1:** Morphometric data at four weeks post-injection. Comparison of mean ± SEM data from control (CON) and monocrotaline-injected (MCT) rats.

	CON (*n* = 11)	MCT (*n* = 10)
Body weight (g)	444 ± 9	386 ± 5 ***
RV–BW (%)	0.36 ± 0.03	0.61 ± 0.02 ***
LV–BW (%)	0.72 ± 0.09	0.89 ± 0.09
Tibial length (mm)	54.51 ± 0.87	51.98 ± 0.54 *
RV–tibial length (%)	2.95 ± 0.22	4.58 ± 0.15 ***
LV–tibial length (%)	6.93 ± 0.30	7.12 ± 0.37
Lung weight (g)	1.80 ± 0.1	2.2 ± 0.1 **
Liver weight (g)	15.40 ± 0.48	14.5 ± 0.03
Lung weight–BW (%)	0.39 ± 0.02	0.56 ± 0.03 ***
Heart rate (min^−1^)	367 ± 23	340 ± 13 **
QTc interval (s)	0.14 ± 0.01	0.15 ± 0.02

RV/LV–BW (%) = right ventricular/left ventricular thickness divided by body weight (BW). QTc = corrected QT interval. *p* values were determined using unpaired, two-tailed *t*-tests, with significance between groups indicated: * *p* < 0.05; ** *p* < 0.01; *** *p* < 0.001 [51].

## References

[1] Naeije R., Manes A. (2014). The right ventricle in pulmonary arterial hypertension. Eur. Respir. Rev..

[2] Ekman I., Cleland J.G., Andersson B., Swedberg K. (2005). Exploring symptoms in chronic heart failure. Eur. J. Heart Fail..

[3] Nieminen M.S., Brutsaert D., Dickstein K., Drexler H., Follath F., Harjola V.-P., Hochadel M., Komajda M., Lassus J., Lopez-Sendon J.L. (2006). EuroHeart Failure Survey II (EHFS II): A survey on hospitalized acute heart failure patients: Description of population. Eur. Heart J..

[4] Maeder M.T., Weber L., Pohle S., Chronis J., Baty F., Rigger J., Brutsche M., Haager P., Rickli H., Brenner R. (2024). Impact of the 2022 pulmonary hypertension definition on haemodynamic classification and mortality in patients with aortic stenosis undergoing valve replacement. Eur. Heart J. Open.

[5] Deng Z., Morse J.H., Slager S.L., Cuervo N., Moore K.J., Venetos G., Kalachikov S., Cayanis E., Fischer S.G., Barst R.J. (2000). Familial primary pulmonary hypertension (gene PPH1) is caused by mutations in the bone morphogenetic protein receptor-II gene. Am. J. Hum. Genet..

[6] Olsson K.M., Corte T.J., Kamp J.C., Montani D., Nathan S.D., Neubert L., Price L.C., Kiely D.G. (2023). Pulmonary hypertension associated with lung disease: New insights into pathomechanisms, diagnosis, and management. Lancet Respir. Med..

[7] Sola J.R., Mena M.S.-C., Riera-Mestre A. (2024). Update in the management of chronic thrombo-embolic pulmonary hypertension. Med. Clin..

[8] Galiè N., Humbert M., Vachiery J.L., Gibbs S., Lang I., Torbicki A., Simonneau G., Peacock A., Vonk Noordegraaf A., Beghetti M. (2016). 2015 ESC/ERS Guidelines for the diagnosis and treatment of pulmonary hypertension: The Joint Task Force for the Diagnosis and Treatment of Pulmonary Hypertension of the European Society of Cardiology (ESC) and the European Respiratory Society (ERS): Endorsed by: Association for European Paediatric and Congenital Cardiology (AEPC), International Society for Heart and Lung Transplantation (ISHLT). Eur. Heart J..

[9] Olivari M.T., Fiorentini C., Polese A., Guazzi M.D. (1978). Pulmonary hemodynamics and right ventricular function in hypertension. Circulation.

[10] Pfeffer J.M., Pfeffer M.A., Fishbein M.C., Frohlich E.D. (1979). Cardiac function and morphology with aging in the spontaneously hypertensive rat. Am. J. Physiol..

[11] Mocumbi A., Humbert M., Saxena A., Jing Z.C., Sliwa K., Thienemann F., Archer S.L., Stewart S. (2024). Pulmonary hypertension. Nat. Rev. Dis. Primers.

[12] Monteagudo-Vela M., Tindale A., Monguió-Santín E., Reyes-Copa G., Panoulas V. (2023). Right ventricular failure: Current strategies and future development. Front. Cardiovasc. Med..

[13] Dignam J.P., Scott T.E., Kemp-Harper B.K., Hobbs A.J. (2022). Animal models of pulmonary hypertension: Getting to the heart of the problem. Br. J. Pharmacol..

[14] Borgdorff M.A., Dickinson M.G., Berger R.M., Bartelds B. (2015). Right ventricular failure due to chronic pressure load: What have we learned in animal models since the NIH working group statement?. Heart Fail. Rev..

[15] Crosby A., Jones F.M., Southwood M., Stewart S., Schermuly R., Butrous G., Dunne D.W., Morrell N.W. (2010). Pulmonary vascular remodeling correlates with lung eggs and cytokines in murine schistosomiasis. Am. J. Respir. Crit. Care Med..

[16] Tsikis S.T., Klouda T., Hirsch T.I., Fligor S.C., Liu T., Kim Y., Pan A., Quigley M., Mitchell P.D., Puder M. (2023). A pneumonectomy model to study flow-induced pulmonary hypertension and compensatory lung growth. Cell Rep. Methods.

[17] Sztuka K., Jasińska-Stroschein M. (2017). Animal models of pulmonary arterial hypertension: A systematic review and meta-analysis of data from 6126 animals. Pharmacol. Res..

[18] Maarman G., Lecour S., Butrous G., Thienemann F., Sliwa K. (2013). A comprehensive review: The evolution of animal models in pulmonary hypertension research; are we there yet?. Pulm. Circ..

[19] Greshoff M. (1890). Mittheilungen aus dem chemisch-pharmakologischen Laboratorium des Botanischen Gartens zu Buitenzorg (Java). Ber. Dtsch. Chem. Ges..

[20] Neal W.M., Rusoff L.L., Ahmann C.F. (1935). The Isolation and Some Properties of an Alkaloid from Crotalaria spectabilis. J. Am. Chem. Soc..

[21] Adams R., Rogers E. (1938). The structure of monocrotaline, the alkaloid in Crotalaria spectabilis and Crotalaria retusa. J. Am. Chem. Soc..

[22] Lalich J., Merkow L. (1961). Pulmonary arteritis produced in rat by feeding Crotalaria spectabilis. Lab. Investig..

[23] Lalich J., Ehrhart L. (1962). Monocrotaline-induced pulmonary arteritis in rats. J. Atheroscler. Res..

[24] Kay J.M., Harris P., Heath D. (1967). Pulmonary hypertension produced in rats by ingestion of Crotalaria spectabilis seeds. Thorax.

[25] D’Alonzo G.E., Barst R.J., Ayres S.M., Bergofsky E.H., Brundage B.H., Detre K.M., Fishman A.P., Goldring R.M., Groves B.M., Kernis J.T. (1991). Survival in patients with primary pulmonary hypertension. Results from a national prospective registry. Ann. Intern. Med..

[26] Wilson D.W., Segall H.J., Pan L.C., Lamé M.W., Estep J.E., Morin D. (1992). Mechanisms and pathology of monocrotaline pulmonary toxicity. Crit. Rev. Toxicol..

[27] Mattocks A., White I. (1971). The conversion of pyrrolizidine alkaloids to N-oxides and to dihydropyrrolizine derivatives by rat-liver microsomes in vitro. Chem. Biol. Interact..

[28] Kasahara Y., Kiyatake K., Tatsumi K., Sugito K., Kakusaka I., Yamagata S.-I., Ohmori S., Kitada M., Kuriyama T. (1997). Bioactivation of monocrotaline by P-450 3A in rat liver. J. Cardiovasc. Pharmacol..

[29] Mattocks A.R. (1968). Toxicity of pyrrolizidine alkaloids. Nature.

[30] Mattocks A.R., White I.N.H. (1971). Pyrrolic metabolites from non-toxic pyrrolizidine alkaloids. Nat. New Biol..

[31] Bruner L.H., Carpenter L.J., Hamlow P., Roth R.A. (1986). Effect of a mixed function oxidase inducer and inhibitor on monocrotaline pyrrole pneumotoxicity. Toxicol. Appl. Pharmacol..

[32] Hanwell A., Linzell J. (1972). Validation of the thermodilution technique for the estimation of cardiac output in the rat. Comp. Biochem. Physiol. A Comp. Physiol..

[33] Pan L.C., Lamé M.W., Morin D., Wilson D.W., Segall H. (1991). Red blood cells augment transport of reactive metabolites of monocrotaline from liver to lung in isolated and tandem liver and lung preparations. Toxicol. Appl. Pharmacol..

[34] E Estep J., Lamé M.W., Morin D., Jones A.D., Wilson D.W., Segall H.J. (1991). [14C]monocrotaline kinetics and metabolism in the rat. Drug Metab. Dispos..

[35] Wilson D.W., Segall H., Pan L.C., Dunston S.K. (1989). Progressive inflammatory and structural changes in the pulmonary vasculature of monocrotaline-treated rats. Microvasc. Res..

[36] Meyrick B., Reid L. (1979). Development of pulmonary arterial changes in rats fed Crotalaria spectabilis. Am. J. Pathol..

[37] Meyrick B., Gamble W., Reid L. (1980). Development of Crotalaria pulmonary hypertension: Hemodynamic and structural study. Am. J. Physiol..

[38] Sardu C., Paolisso G., Marfella R. (2020). Inflammatory Related Cardiovascular Diseases: From Molecular Mechanisms to Therapeutic Targets. Curr. Pharm. Des..

[39] Hilliker K.S., Bell T.G., Roth R.A. (1982). Pneumotoxicity and thrombocytopenia after single injection of monocrotaline. Am. J. Physiol..

[40] Buermans H.P.J., Redout E.M., Schiel A.E., Musters R.J.P., Zuidwijk M., Eijk P.P., van Hardeveld C., Kasanmoentalib S., Visser F.C., Ylstra B. (2005). Microarray analysis reveals pivotal divergent mRNA expression profiles early in the development of either compensated ventricular hypertrophy or heart failure. Physiol. Genom..

[41] Mathew R., Altura B., Altura B. (1989). Strain differences in pulmonary hypertensive response to monocrotaline alkaloid and the beneficial effect of oral magnesium treatment. Magnesium.

[42] Goldenthal E.I., D’Aguanno W., Lynch J.F. (1964). Hormonal Modification of the Sex Differences Following Monocrotaline Administration. Toxicol. Appl. Pharmacol..

[43] Tofovic P.S., Zhang X., Petrusevska G. (2009). Progesterone inhibits vascular remodeling and attenuates monocrotaline-induced pulmonary hypertension in estrogen-deficient rats. Prilozi.

[44] Lookin O., Kuznetsov D., Protsenko Y. (2015). Sex differences in stretch-dependent effects on tension and Ca(2+) transient of rat trabeculae in monocrotaline pulmonary hypertension. J. Physiol. Sci..

[45] Silveyra P., Fuentes N., Bauza D.E.R. (2021). Sex and Gender Differences in Lung Disease. Adv. Exp. Med. Biol..

[46] Nogueira-Ferreira R., Vitorino R., Ferreira R., Henriques-Coelho T. (2015). Exploring the monocrotaline animal model for the study of pulmonary arterial hypertension: A network approach. Pulm. Pharmacol. Ther..

[47] Lai Y.L., Olson J.W., Gillespie M.N., Gomez-Arroyo J.G., Farkas L., Alhussaini A.A., Farkas D., Kraskauskas D., Voelkel N.F., Bogaard H.J. (1991). Ventilatory dysfunction precedes pulmonary vascular changes in monocrotaline-treated rats. J. Appl. Physiol..

[48] Hardziyenka M., Campian M.E., de Bruin-Bon H.R., Michel M.C., Tan H.L. (2006). Sequence of echocardiographic changes during development of right ventricular failure in rat. J. Am. Soc. Echocardiogr..

[49] Power A.S. (2017). Mitochondrial Energy Transfer in Animal Models of Left and Right Ventricular Hypertrophy: Investigating Mitochondrial Oxidative Phosphorylation and Calcium Handling in the Heart and Their Contribution to Contractile Dysfunction in Cardiac Hypertrophy. Ph.D. Thesis.

[50] Power A.S., Hickey A.J., Crossman D.J., Loiselle D.S., Ward M.-L. (2018). Calcium mishandling impairs contraction in right ventricular hypertrophy prior to overt heart failure. Pflug. Arch..

[51] Krstic A.M., Kaur S., Ward M.-L. (2020). Response of non-failing hypertrophic rat hearts to prostaglandin F2α. Curr. Res. Physiol..

[52] Krstic A.M., Power A.S., Ward M.-L. (2023). Increased Mitochondrial Calcium Fluxes in Hypertrophic Right Ventricular Cardiomyocytes from a Rat Model of Pulmonary Artery Hypertension. Life.

[53] Lookin O., Kuznetsov D., Protsenko Y. (2022). Omecamtiv mecarbil attenuates length-tension relationship in healthy rat myocardium and preserves it in monocrotaline-induced pulmonary heart failure. Clin. Exp. Pharmacol. Physiol..

[54] Fowler E.D., Hauton D., Boyle J., Egginton S., Steele D.S., White E. (2019). Energy Metabolism in the Failing Right Ventricle: Limitations of Oxygen Delivery and the Creatine Kinase System. Int. J. Mol. Sci..

[55] Han J.-C., Guild S.-J., Pham T., Nisbet L., Tran K., Taberner A.J., Loiselle D.S. (2017). Left-Ventricular Energetics in Pulmonary Arterial Hypertension-Induced Right-Ventricular Hypertrophic Failure. Front. Physiol..

[56] Fowler E.D., Benoist D., Drinkhill M.J., Stones R., Helmes M., Wüst R.C., Stienen G.J., Steele D.S., White E. (2015). Decreased creatine kinase is linked to diastolic dysfunction in rats with right heart failure induced by pulmonary artery hypertension. J. Mol. Cell. Cardiol..

[57] Krstic A.M., Power A.S., Ward M.-L. (2022). Visualization of Dynamic Mitochondrial Calcium Fluxes in Isolated Cardiomyocytes. Front. Physiol..

[58] Pham T., Loiselle D., Power A., Hickey A.J.R. (2014). Mitochondrial inefficiencies and anoxic ATP hydrolysis capacities in diabetic rat heart. Am. J. Physiol. Cell Physiol..

[59] Power A.S., Norman R., Jones T.L.M., Hickey A.J., Ward M.-L. (2019). Mitochondrial function remains impaired in the hypertrophied right ventricle of pulmonary hypertensive rats following short duration metoprolol treatment. PLoS ONE.

[60] Pham T., Nisbet L., Taberner A., Loiselle D., Han J. (2018). Pulmonary arterial hypertension reduces energy efficiency of right, but not left, rat ventricular trabeculae. J. Physiol..

[61] Korstjens I., Rouws C., Van Der Laarse W., Van Der Zee L., Stienen G. (2002). Myocardial force development and structural changes associated with monocrotaline induced cardiac hypertrophy and heart failure. J. Muscle Res. Cell Motil..

[62] Kögler H., Hartmann O., Leineweber K., Nguyen van P., Schott P., Brodde O.E., Hasenfuss G. (2003). Mechanical load-dependent regulation of gene expression in monocrotaline-induced right ventricular hypertrophy in the rat. Circ. Res..

[63] Lamberts R.R., Hamdani N., Soekhoe T.W., Boontje N.M., Zaremba R., Walker L.A., De Tombe P.P., Van Der Velden J., Stienen G.J. (2007). Frequency-dependent myofilament Ca^2+^ desensitization in failing rat myocardium. J. Physiol..

[64] Miura M., Hirose M., Endoh H., Wakayama Y., Sugai Y., Nakano M., Fukuda K., Shindoh C., Shirato K., Shimokawa H. (2011). Acceleration of Ca^2+^ waves in monocrotaline-induced right ventricular hypertrophy in the rat. Circ. J. Off. J. Jpn. Circ. Soc..

[65] Wüst R.C., de Vries H.J., Wintjes L.T., Rodenburg R.J., Niessen H.W., Stienen G.J. (2016). Mitochondrial complex I dysfunction and altered NAD(P)H kinetics in rat myocardium in cardiac right ventricular hypertrophy and failure. Cardiovasc. Res..

[66] Werchan P.M., Summer W.R., Gerdes A.M., McDonough K.H. (1989). Right ventricular performance after monocrotaline-induced pulmonary hypertension. Am. J. Physiol..

[67] Vescovo G., Harding S.E., Jones M., Libera L.D., Pessina A.C., Poole-Wilson P.A. (1989). Contractile abnormalities of single right ventricular myocytes isolated from rats with right ventricular hypertrophy. J. Mol. Cell. Cardiol..

[68] Honda M., Kuramochi T., Ishinaga Y., Kuzuo H., Tanaka K., Morioka S., Enornoto K.I., Takabatake T. (1994). Contrasting effects of isoproterenol and phosphodiesterase III inhibitor on intracellular calcium transients in cardiac myocytes from failing hearts. Clin. Exp. Pharmacol. Physiol..

[69] Benoist D., Stones R., Drinkhill M.J., Benson A.P., Yang Z., Cassan C., Gilbert S.H., Saint D.A., Cazorla O., Steele D.S. (2012). Cardiac arrhythmia mechanisms in rats with heart failure induced by pulmonary hypertension. Am. J. Physiol. Heart Circ. Physiol..

[70] Umar S., Lee J.-H., de Lange E., Iorga A., Partow-Navid R., Bapat A., van der Laarse A., Saggar R., Saggar R., Ypey D.L. (2012). Spontaneous ventricular fibrillation in right ventricular failure secondary to chronic pulmonary hypertension. Circ. Arrhythm. Electrophysiol..

[71] Benoist D., Stones R., Drinkhill M.J., Bernus O., White E., Natali A.J., Fowler E.D., Calaghan S.C., Benson A.P., Yang Z. (2011). Arrhythmogenic substrate in hearts of rats with monocrotaline-induced pulmonary hypertension and right ventricular hypertrophy. Am. J. Physiol. Heart Circ. Physiol..

[72] Bers D.M. (2002). Cardiac excitation-contraction coupling. Nature.

[73] Fabiato A. (1983). Calcium-induced release of calcium from the cardiac sarcoplasmic reticulum. Am. J. Physiol..

[74] Lee J.-K., Kodama I., Honjo H., Anno T., Kamiya K., Toyama J., Benoist D., Stones R., Drinkhill M.J., Benson A.P. (1997). Stage-dependent changes in membrane currents in rats with monocrotaline-induced right ventricular hypertrophy. Am. J. Physiol..

[75] Power A., Kaur S., Dyer C., Ward M.-L. (2020). Disruption of Transverse-Tubules Eliminates the Slow Force Response to Stretch in Isolated Rat Trabeculae. Front. Physiol..

[76] Bristow M.R., Minobe W., Rasmussen R., Larrabee P., Skerl L., Klein J.W., Anderson F.L., Murray J., Mestroni L., Karwande S.V. (1992). Beta-adrenergic neuroeffector abnormalities in the failing human heart are produced by local rather than systemic mechanisms. J. Clin. Investig..

[77] Krstic A., Ward M.-L. (2021). Ca^2+^ Handling in Non-Failing Hypertrophic Cardiomyocytes Subjected to Inotropic Interventions. Biophys. J..

[78] Jonigk D., Golpon H., Bockmeyer C.L., Maegel L., Hoeper M.M., Gottlieb J., Nickel N., Hussein K., Maus U., Lehmann U. (2011). Plexiform lesions in pulmonary arterial hypertension composition, architecture, and microenvironment. Am. J. Pathol..

[79] Ruiter G., de Man F.S., Schalij I., Sairras S., Grünberg K., Westerhof N., van der Laarse W.J., Vonk-Noordegraaf A. (2013). Reversibility of the monocrotaline pulmonary hypertension rat model. Eur. Respir. J..

[80] Faber M.J., Dalinghaus M., Lankhuizen I.M., Steendijk P., Hop W.C., Schoemaker R.G., Duncker D.J., Lamers J.M.J., Helbing W.A. (2006). Right and left ventricular function after chronic pulmonary artery banding in rats assessed with biventricular pressure-volume loops. Am. J. Physiol. Heart Circ. Physiol..

[81] Hessel M.H.M., Steendijk P., Adel B.D., Schutte C.I., van der Laarse A. (2006). Characterization of right ventricular function after monocrotaline-induced pulmonary hypertension in the intact rat. Am. J. Physiol. Heart Circ. Physiol..

